# Le recours systématique à l’estimation échographique du poids fœtal en salle de naissances augmente-t-il le risque d’accouchement par césarienne?

**DOI:** 10.11604/pamj.2016.25.57.8880

**Published:** 2016-09-30

**Authors:** Kaouther Dimassi, Meryam Ajroudi, Olfa Saidi, Safa Salem, Monia Robbana, Amel Triki, Mohammed Faouzi Gara

**Affiliations:** 1 Service de Gynécologie-Obstétrique, Hôpital Mongi Slim La Marsa, Tunisie; 2 Université Tunis El Manar, Faculté de Médecine de Tunis, Tunisie

**Keywords:** Estimated fetal weight, ultrasound, cesarean delivery, birth weight, Fetal weight estimation (FWE), ultrasound, caesarean, weight at birth

## Abstract

L’échographie est un précieux outil utilisé quotidiennement en salle de travail. Ses applications sont multiples. L’objectif est d’évaluer si le recours systématique à une estimation échographique du poids fœtal et en salle de naissances augmente le risque d’accouchement par césarienne. Etude de cohorte monocentrique. Nous avons inclus toutes les parturientes avec des grossesses monofœtales qui avaient accouché à un terme ≥ 39 SA. Nous avons exclu toutes les patientes avec une contre indication à l'accouchement par voie basse ainsi que celles chez lesquelles une Estimation échographique du poids fœtal (EEPF) le jour de l'accouchement était jugée nécessaire à la prise de décision obstétricale. Les parturientes ainsi retenues ont été réparties en deux groupes: - G1 : parturientes ayant systématiquement eu une EEPF - G2: parturientes n’ayant pas eu cette EEPF. Nous avons comparé le taux d’accouchement par césarienne avec ajustement sur les potentiels facteurs confondants selon la régression logistique. 838 parturientes ont été retenues pour ce travail. La primiparité, l'EEPF ainsi que le poids à la naissance étaient des facteurs de risque d'accouchement par césarienne. Après ajustement aux facteurs confondants, l'EEFP réalisée systématiquement dans G1 s'est révélé être un facteur de risque indépendant d'accouchement par césarienne avec un OR =3,8 (IC95% = [2,67-5,48]). Ce risque augmentait significativement avec le poids estimé (PFE): OR=2,27(IC95;1,15-4,47;p=0.018) pour 3500 < PFE< 4000g et OR= 10,64(IC95; 4,28-26,41;p<0.001) pour PFE>4000g. L’EEPF réalisée systématiquement en salle de naissances représente un facteur de risque indépendant et potentiellement modifiable d’accouchement par césarienne.

## Abstract (en)

Ultrasound is a valuable tool commonly used in the delivery room. It has multiple applications. The objective of this study was to investigate whether systematic fetal weight estimation by ultrasound in the delivery room increases the risk of cesarean delivery. Monocentric cohort study. All parturients with singleton pregnancies who gave birth full-term at = 39 weeks were enrolled in the study. We excluded all patients with a contraindication to vaginal birth as well as those in whom fetal weight estimation (FWE) by ultrasound on day of delivery was deemed necessary in making obstetric decision. Parturients enrolled in the study were divided into two groups: - G1: parturients who systematically underwent FWE - G2: parturients who never underwent FWE. We compared cesarean delivery rate with adjustment for potentially confounding factors according to logistic regression. 838 parturients were enrolled in the study. Prematurity, FWE and weight at birth were risk factors for cesarean delivery. After adjustment for confounding factors, FWE by ultrasound systematically performed in G1 proved to be an independent risk factor for cesarean delivery with OR = 3.8 (CI 95% = [2.67 to 5.48]). This risk increased significantly with estimated fetal weight (EFW): OR=2,27(CI 95;1,15-4,47; p=0.018) for 3500 < EFW < 4000g and OR = 10.64 (CI 95; 4.28 to 26.41; p < 0.001 ) for EFW > 4000 g. FWE by ultrasound systematically performed in the delivery room represents an independent and potentially modifiable risk factor for cesarean delivery.

## Introduction

L’échographie est un précieux outil utilisé quotidiennement en obstétrique et communément utilisé en salle de travail. Ses applications sont multiples. Elle vient essentiellement compléter l’examen clinique en cas de difficulté [1]. L’une de ses principales applications réside dans l’estimation échographique du poids fœtal (EEPF) [2]. La performance de cette dernière a largement été discutée dans la littérature [2, 3]. Actuellement, le recours à l’EEPF en salle de naissance doit être facilité lorsque la situation clinique s´y prête par exemple en cas de suspicion clinique de macrosomie. Ce ci peut être critiqué d’autant plus que cet examen échographique a tendance à se substituer à l’estimation clinique. De ce fait l’EPF en salle de naissance demeure controversée. Dans ce sens, nous nous proposons de voir si l’EEPF systématiquement réalisée dans ces conditions peut représenter un facteur de risque indépendant d‘accouchement par césarienne.

## Méthodes

Il s’agit d’une étude de cohorte monocentrique et rétrospective menée dans une maternité tunisienne de niveau 2b sur une période de un an (de janvier à décembre 2014). Cette étude a été approuvée par le comité d´éthique de l´hôpital Mongi Slim, La Marsa. Nous avons inclus les observations de toutes les parturientes avec des grossesses monofœtales qui avaient accouché dans notre maternité à un terme supérieur ou égal à 39 semaines d’aménorrhée (SA). Ce terme a été choisi pour deux raisons : premièrement pour exclure tous les cas ayant nécessité un accouchement avant 39 SA pour une cause maternelle ou fœtale et deuxièmement car au delà de 39 SA, le risque d’accouchement dystocique est plus important du fait d’un poids fœtal plus élevé par rapport à un terme plus précoce. Dans ce sens, l’estimation échographique aura plus d’impact sur la prise de décision. Par la suite, nous avons exclu les observations des parturientes chez lesquelles une EPF réalisée le jour de l’accouchement et en salle de travail, était nécessaire à la prise de décision obstétricale. En exemple: les parturientes ayant un utérus uni cicatriciel, ou avec une présentation de siège à terme, ou encore celles prises en charge pour une dysgravidie telle qu’un diabète gestationnel, une prééclampsie, un retard de croissance intra-utérin… De plus, nous avons exclu les parturientes avec une décision d’accouchement par une césarienne programmée. En exemple : les patientes avec un placenta prævia recouvrant, les patientes avec une contre indication à l’accouchement par voie basse…) Une fiche de recueil de données a été remplie pour chacune des observations retenues pour l’étude. Les informations suivantes étaient systématiquement relevées à partir du dossier obstétrical : l’âge, la parité, le terme gestationnel, l’index de masse corporelle (IMC) calculé le jour de l’accouchement, l’éventuelle réalisation systématique d’une EEPF en salle de travail, le poids fœtal estimé échographiquement (PFEE), le poids fœtal estimé cliniquement par la manœuvre de Léopold [4], la mesure de la hauteur utérine (HU), le mode d’accouchement, l’indication d’une éventuelle césarienne ainsi que l’Apgar et le poids à la naissance (PN).

Par la suite, les parturientes retenues pour l’étude ont été réparties en deux groupes: *un premier groupe (G1)*de parturientes ayant systématiquement eu une EEPF le jour de leur admission en salle de travail pour accouchement ; *un deuxième groupe (G2)* de parturientes n’ayant pas eu cette EEPF.

Chez toutes les patientes du G1, l’examen échographique était réalisé au moyen d’un échographe : TOSHIBA modèle SSA-510A, muni d’une sonde abdominale de 5-2 MHrz. Le PFE était calculé à l’aide de la formule de Hadlock C [5] (Log10 EPF = 1,335+0.0316 BIP+ 0,0457 PA + 0,1623 LF – 0,0034 PA LF). L’obésité maternelle était définie par un IMC > 30kg/m^2^ en début de grossesse ou à 35kg/m^2^ à terme [2]. Toutes, les données ont été recueillies dans un tableau EXCEL 2007 et l’analyse statistique a été réalisée par le logiciel XLSTAT (2014.4.09; Addinsoft, New York, NY, USA). Les variables quantitatives sont données en en moyennes +/- l’écart type.

Les variables qualitatives sont données avec le nombre et le pourcentage pour chaque catégorie. La comparaison entre les groupes a été effectuée par un test de Chi^2^ pour les variables qualitatives et par le test de Student pour les variables continues. Les différences étaient considérées comme statistiquement significatives si p <0.05. Dans un premier temps, nous avons comparé le taux d’accouchement par césarienne (toutes indications confondues) entre les deux groupes. Afin de voir si l’EPF représente un facteur de risque indépendant d’accouchement par césarienne, nous avons utilisé la régression logistique. L’événement aléatoire était le mode d’accouchement.

Dans un deuxième temps et pour les patientes de G1, nous avons étudié la performance de l’EEPF. Dans ce sens, les différences entre les PFEE et les PN ont été calculées en termes de différence absolue (PFEE-PN) et de pourcentage d’erreur ((PFEE-PN)*100/PN). Nous avons réalisé une étude de la corrélation entre PFEE et PN en utilisant la régression linéaire de Pearson. ainsi qu´une étude de concordance selon la méthode de Bland et Altman [6]. Enfin, nous avons étudié la performance de l’EEPF dans le diagnostic de macrosomie (définie par un PN ≥4000gr [7]) et pour le diagnostic de macrosomie extrême (définie par un PN ≥4500gr [8]). Dans ce sens, nous avons calculé la sensibilité, la spécificité, la valeur prédictive positive (VPP) ainsi que la valeur prédictive négative (VPN) de l’examen échographique pour chacune de ces situations.

## Résultats

**Figure 1:**
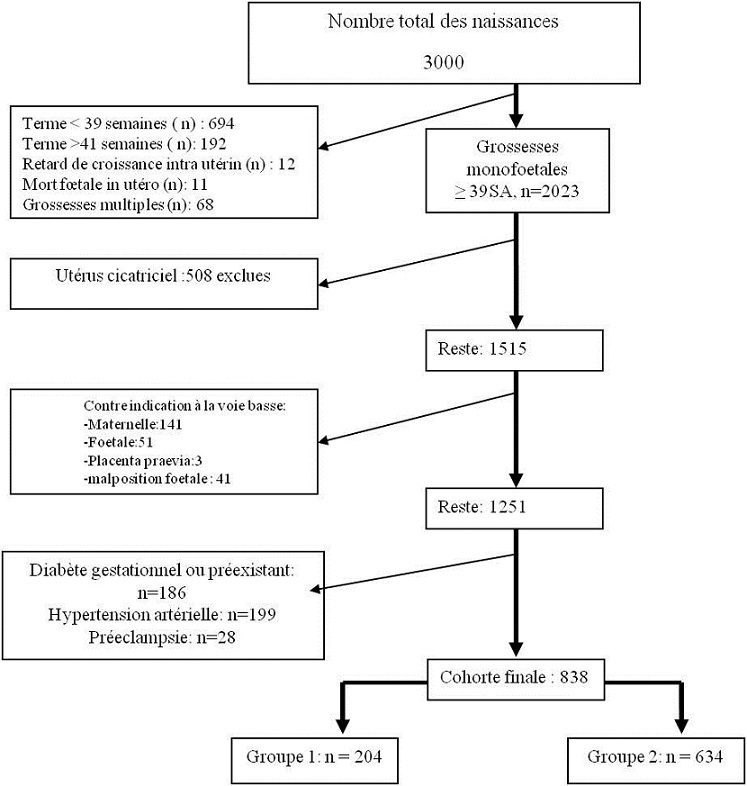
Critères d’inclusion et d’exclusion des parturientes

Durant la période d’étude, 3000 femmes ont accouché dans notre service dont 838 ont été retenues pour cette étude. La Figure 1 détaille les motifs de non inclusion et d’exclusion des 2162 autre parturientes. Deux cents quatre parturientes (24,4%) avaient eu une EEPF au cours de leur hospitalisation pour accouchement et ont ainsi été classées dans G1. Les six cents trente quatre parturientes n’ayant pas eu d’EEPF et ont été inclues dans G2.

Le Tableau 1 résume les caractéristiques épidémiologiques des deux groupes de patientes. Le taux de primiparité était significativement plus élevé dans G1: 69,9% Vs 49% dans G2 avec p <0,001. De même, l’âge gestationnel et le poids à la naissance étaient significativement plus élevés dans G1. Par ailleurs, nous n’avons pas objectivé de différence significative en termes d’âge maternel et de taux d’obésité.

**Tableau 1:** Caractéristiques épidémiologiques des deux groupes de patientes

	**G1**	**G2**	**P**
Age moyen (moyenne/écart type)	29.16 s =4.965	29.26 s = 4.705	0.32
IMC Kg/m² (moyenne/écart type)	29.8 s=3.540	29.2 s = 3.698	0.75
Obésité (%)	9.3	7.2	0.7
Parité (moyenne/écart type)	1.48 s=0.839	1.74 s= 0.876	<0.001
Taux de primiparité (%)	69.1	49	<0.001
Hauteur uterine (moyenne en cm /écart type)	32.28 s=2.316	31.63 s =2.200	0.001
Âge gestationnel moyen (SA) (moyenne en cm /écart type)	40.1 s=0.817	39.8 s = 0.732	<0.001
Poids de naissance moyen (g) (moyenne en cm /écart type)	3555,57 s= 529,148	3430,44 s=430,857	0.001

Le taux d´accouchement par césarienne était significativement plus élevé dans G1 : 54,7 % Vs 19,7 % (p < 0,001) (voir le Tableau 2). Nous avons objectivé que la primiparité, l´EEPF ainsi que le poids à la naissance étaient des facteurs de risque d´accouchement par césarienne (voir le Tableau 3). Après ajustement aux facteurs confondants, l´EEPF réalisée systématiquement dans G1 s´est révélé être un facteur de risque indépendant d´accouchement par césarienne avec un OR =3,8; IC 95% = [2,67-5,48] (voir le Tableau 3). Ce risque était particulièrement augmenté pour un PFEE ≥ 4000gr avec un OR ajusté à 10.64 ; IC 95% = [4,28-26,41] (voir le Tableau 4).

**Tableau 2:** Caractéristiques respectives des modalités du travail et de l'accouchement dans les deux groupes de parturientes

	G1	G2	P
Direction du travail n (%)	203 (59.1)	629 (64.1)	0.2
Voie basse n (%)	92 (45.3)	509 (80.3)	< 0.001
Césarienne n (%)	111 (54.7)	125 (19.7)	
Césarienne pour souffrance fœtale aigue n (%)	37 (18.04)	80 (12.6)	-
Taux de césarienne pour macrosomie n (%)	25 (12.3)	6 (0.9)	-
Taux de césarienne pour défaut d’engagement n (%)	15 (7.4)	9 (1.4)	-
Taux de césarienne pour dystocie de			
Démarrage n (%)	4 (2)	2 (0.3)	-
Autre indication de césarienne n (%)	30 (14.9)	5 (4.5)	-

**Tableau 3:** Facteurs de risque d’accouchement par césarienne

	Modèle 1+ OR Brut [IC]	Modèle 2++ OR Ajusté [IC]	P (Wald’s)
Estimation échographique	4.91 [3.5,6.88]	**3.82 [2.67,5.48]**	**< 0.001**
Poids naissance			
<3500			**< 0.001**
3500-4000	1.68 [1.21,2.34]	1.63 [1.13,2.35]	
≥4000	2.16 [1.36,3.43]	2.57 [1.5,4.42]	
Primiparité	4.57 [3.23,6.49]	4.5 [3.09,6.55]	**< 0.001**

+ Modèle 1: association brute

++ Modèle 2 : associations ajustées sur les facteurs (Estimation échographique, poids réel à la naissance et primiparité) ; OR : Odds-ratio de la catégorie par rapport à la catégorie de référence ; IC : Intervalle de confiance (P=0,95)

**Tableau 4:** Ajustement du risque de césarienne en fonction du poids estimé

	Modèle 1^+^OR Brut [IC]	Modèle 2^++^OR Ajusté [IC]	P(Wald’s)
Poids estimé <3500g	Référence		
Poids estimé 3500- 4000g	2.24 (1.17,4.3)	2.27 (1.15,4.47)	0.018
Poids estimé ≥4000g	6.93 (3.07,15.63)	10.64 (4.28,26.41)	<0.001

**Figure 2:**
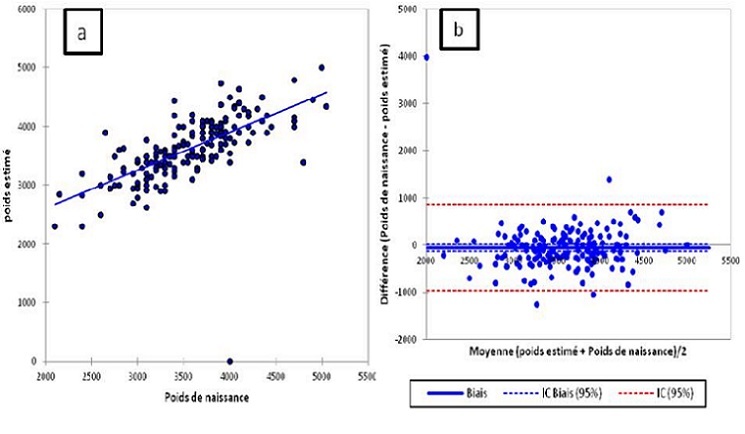
Etude de la pertinence de l’EEPF systématiquement réalisée dans G1. A) étude de corrélation; B) étude de concordance selon Bland et Altmann

En ce qui concerne l´étude de la pertinence de l´EEPF, la différence moyenne entre le PFEE et PN était de 264g [0-1250]. La marge d´erreur moyenne en valeur absolue était de 7,96% [0,59-32] avec 74,58% des estimations ayant une marge d´erreur inférieure à 10% en valeur absolue. Nous avons objectivé une corrélation positive et significative entre les deux valeurs avec R = 0'>58% des estimations ayant une marge d´erreur inférieure à 10% en valeur absolue. Nous avons objectivé une corrélation positive et significative entre les deux valeurs avec R = 0,64 et p < 10^-3^. Enfin, l´étude de concordance avait mis en évidence un biais systématique de l´étude de concordance avait mis en évidence un biais systématique de -50,619g; IC 95%619g ; IC 95% ]-118,698;17,460[. Les limites de concordances étaient importantes'>. Les limites de concordances étaient importantes ]-960,372; 859,134[ (voir la Figure 2).

## Discussion

Cette étude, a été réalisée sur un nombre conséquent de patientes. En réalité nous avons inclus toutes les parturientes ayant accouché dans notre unité durant une année. Le caractère mono centrique permet de garantir l’uniformité des conduites et des indications de césarienne. De plus'> a été réalisée sur un nombre conséquent de patientes. En réalité nous avons inclus toutes les parturientes ayant accouché dans notre unité durant une année. Le caractère mono centrique permet de garantir l’uniformité des conduites et des indications de césarienne. De plus, nous avons exclus toutes les patientes chez lesquelles l’examen échographique trouvait son indication en salle de naissance. La principale discussion étant ici l’impact des EEPF itératives. Les résultats mettent clairement en évidence un sur'> nous avons exclus toutes les patientes chez lesquelles l’examen échographique trouvait son indication en salle de naissance. La principale discussion étant ici l’impact des EEPF itératives. Les résultats mettent clairement en évidence un sur-risque de césarienne en cas d’EEPF itérative. Ce risque persiste après régression logistique et exclusion de tous les facteurs confondants comme la primiparité et le poids de naissance avec OR = 3,82 (IC 95% = [2'>82 (IC 95% = [2,67-5,48]). Nous pouvons déduire que la connaissance préalable du poids fœtal peut influer sur la prise de décision de l’obstétricien et de la parturiente d’autant plus que le sur-risque de césarienne augmente avec la valeur du poids estimé : OR= 2,27 (IC95=1,15-4,47;p=0.018) pour 3500g < PFEE < 4000g et OR =10.64 (4,28-26,41;p<0,001) pour un PFEE ≥4000g. Le caractère rétrospectif demeure la principale limite à attribuer à ce travail et afin d’avoir des résultats plus puissants il faudrait organiser une étude similaire sur le mode prospectif et randomisé.

Cette discussion a déjà fait le sujet d’autres publications. Il a été démontré dans ces études que l’échographie itérative en salle de naissance augmente considérablement et indépendamment des autres facteurs le risque de recours à la césarienne. En exemple, Little SE et al. [1] attribuent à l’échographie un sur-risque d’accouchement par césarienne avec un OR global de 1,44 et OR de 1,8 quand le PFEE est > 3500g. Cependant, dans ce travail rétrospectif les patientes avec un utérus cicatriciel n’ont pas été exclues. Dans notre pratique, l’EEPF est indiquée chez les patientes avec un utérus cicatriciel et l’épreuve utérine est acceptée pour un PFE ≤4500g [9]. De plus, vu le caractère multicentrique de l’étude de Little SE et al., la non universalité du codage des indications de césarienne ou des échographies a été discutée en tant que facteur limitant. Encore une fois, dans notre étude monocentrique avec un nombre limité d’intervenants, ces difficultés n’ont pas été rencontrées.

Ainsi, le caractère monocentrique de cette étude a permis l’uniformité des conduites, l’utilisation d’un même appareillage et la réalisation de ce travail au sein d’une même équipe. Ces critères n’ont pas été exigés dans les autres études contrairement à la notre. Le but était d’éliminer encore tout facteur pouvant constituer un biais de sélection. Nos résultats ont montré que l’échographie est un facteur de risque indépendant d’accouchement par césarienne.

L’étude de la pertinence de l’estimation échographique réalisée dans notre étude avait mis en évidence des résultats concordants à ceux précédemment publiés. En effet, différence moyenne entre le PFEE et PN était de 264g [0-1250]. La marge d´erreur moyenne en valeur absolue était de 7,96% [0,59-32] avec 74,58% des estimations ayant une marge d´erreur inférieure à 10% en valeur absolue. Ces résultats rejoignent ceux publiés par la même équipe lors d’un travail prospectif réalisé sur 500 EEPF [10]. Nous retenons les mêmes limites à cette estimation devant des limites de concordances importantes'> Nous retenons les mêmes limites à cette estimation devant des limites de concordances importantes ]-960,372; 859,134 [2]. Ainsi, le sur-risque de césarienne attribuable l’EEPF ne semble pas être lié à une performance particulièrement moins bonne que ce qui a été précédemment démontré.

D’une manière plus précise le risque augmente en cas de suspicion de macrosomie (PFE ≥ 4kg) ou de macrosomie extrême (PFE >4500g). Ceci nous amène à réfléchir sur nos conduites et nos prises de décisions d’autant plus qu’en réalité la sensibilité de l’échographie pour le diagnostic de ces anomalies est connue pour être faible [11]. Les paramètres cliniques comme l’accommodation foeto-pelvienne ainsi que l’évolution du travail représentent des critères logiques de prise de décision mais ne protègent pas toujours contre les situations de dystocie des épaules. Par contre, l’échographie permet de limiter au mieux le risque de poids de naissance supérieur à 4250 g et donc de sélectionner une population à risque non majoré de dystocie des épaules [12]. A l’heure où l’échographie a clairement gagné sa place en salles de naissances et où elle vient pallier aux insuffisances de l’examen clinique en deuxième phase de travail comme pour le diagnostic d’engagement [3] ou encore pour la détermination de la variété de position [13] Serait-il anodin d’y avoir recours d’une manière systématique? De même, la survenue d’une dystocie des épaules avec une macrosomie suspectée échographiquement le jour de l’accouchement serait-elle préjudiciable sur le plan médico-légal? D’un autre coté, le contrôle du taux de césarienne représente une préoccupation importante dans le domaine obstétrical [14–16] et actuellement, la crainte du risque obstétrical aboutit à une surmédicalisation de l’accouchement et à l’évolution du taux de césariennes sur le mode pandémique.

Les résultats de ce travail appuient la thèse que l’échographie en salle de naissances peut représenter un risque indépendant et en conséquences potentiellement modifiable d’accouchement par césarienne. Le recours à un examen échographique en salle de naissances devra avoir ses indications. L’apport indéniable dans le diagnostic de variété de position ou d’engagement ne peut pas masquer les limites attribuables à l’estimation du poids fœtal.

## Conclusion

L’estimation échographique systématique du poids fœtal réalisée en salle de naissance représente un facteur de risque indépendant d’accouchement par césarienne. Ainsi, et comme tout examen complémentaire, le recours à l’échographie en salles de naissances devra pouvoir être justifié.

### Etat des connaissances actuelles sur le sujet

- L’échographie itérative en salle de naissance augmente considérablement le risque de recours à la césarienne tout facteur confondu;
- L’échographie est un facteur de risque indépendant aussi de recours à la césarienne en salle de naissance;
- Le recours à l’échographie en salle de naissance doit être justifié et non systématique.

### Contribution de notre étude à la connaissance

- Le caractère monocentrique a permis l’uniformité des conduites;
- L’utilisation d’un même appareillage et la réalisation de ce travail au sein d’une même équipe;
- Ces critères n’ont pas été exigés dans les autres études contrairement à la notre. Le but était d’éliminer encore tout facteur pouvant constituer un biais de sélection.
